# Supplementary material for: STRAP regulates alternative splicing fidelity during lineage commitment of mouse embryonic stem cells
Source: Nat Commun. 2020 Nov 23;11:5941. doi: 10.1038/s41467-020-19698-6 (PMC7684319; doi:10.1038/s41467-020-19698-6)
Supplement: Supplementary file 1 — Supplementary Information [file 41467_2020_19698_MOESM1_ESM.pdf]

STRAP regulates alternative splicing fidelity during lineage commitment of mouse embryonic stem cells

Lin Jin et al, Supplementary information.

Supplementary Figure 1

a

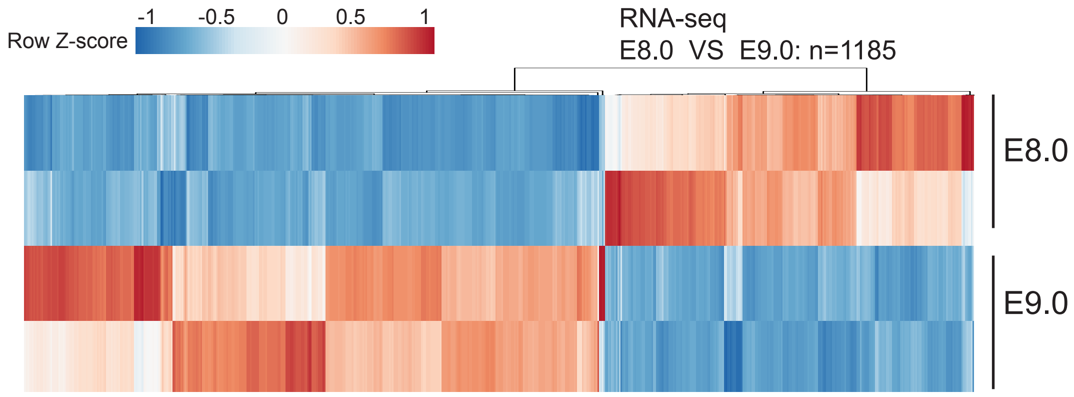

Upregulation in E8.0 mouse embryo: 473 genes

GO Function	Representative Genes
utero embryonic development	*Slc34a2, Hhex, Hes3, Gata4, Dab2, Cdh1*
neural tube closure	*St14, Spint1, Sdc4, Sall1, Pax2, Lrp2, Grhl3*
immune system process	*Sema4a, Ptk2b, Mcoln2, Ifitm1, Fgg, Fgb*
mitochondria	*Prodh2, Dmgdh, Tfcp2l1, Tdh, Slc16a1, Prodh2, Dmgdh*
metal ion binding	*Sp7, Sp5, Smad9, Slc6a4, Sall1, S100a1, Nt5e*
transcription factor complex	*Tfcp2l1, Smad9, Pou5f1, Pax2, Foxh1, Hes7, Hes3, Klf5, Foxa3, Foxa1*
nervous system development	*Nrg1, Lrp2, Lhx1, Hoxa1, Gfra2, Fos, Epha5, Apob*
heart development	*Gata4, Tdgf1, Myocd, Mmp9, Fgf15*
negative regulation of apoptotic process	*Epha5, Tdgf1, Ptk2b, Plac8, Pax2*
cell projection	*Rasgrp2, Rab25, Rab17, Ptk2b, Podxl, Plekhg6, Pkd1l1, Myo6*
neuronal cell body	*Kcnc3, Rab17, Ptk2b, Myo6, Kiss1, Hpn, Epha5, Ass1*
stem cell differentiation	*Zscan10, Pou5f1, Lin28a, Etv4*

Upregulation in E9.0 mouse embryo: 712 genes

GO Function	Representative Genes
skeletal system development	*Col5a2, Col2a1, Trp63, Pitx1, Pax1, Hoxa10, Col5a2, Col2a1*
calcium ion binding	*Spock2, Cdh8, Tll1, Thbs2, Tbc1d9, Spta1, Nell2, Dchs1*
blood vessel development	*Sphk1, Lox, Gja4, Col1a2, Antxr1, Nrarp, Sfrp2, Plxnd1*
Wnt signaling pathway	*Wnt9a, Wnt4, Wnt7a, Wnt7b, Wnt8b, Tnik, Sostdc1, Sfrp2, Rspo1, Pygo1*
transcription factor complex	*Nfatc4, Dach2, Twist2, Tbx2, Pou3f2, Pitx1, Pax1, Olig2, Nkx2-1*
Golgi apparatus	*Trp63, Tenm2, Stmn3, St8sia1, Sptb, Slc30a10, Rab27b, Postn, Plk3, Plagl1, Parm1*
Notch signaling pathway	*Hes5, Hes6. Trp63. Tbx2, S1pr3, Nrarp, Jag2, Cntn1, Cebpa*
nervous system development	*Gng8, Nav3, Vax1, Uncx, Tubb2b, Tnik, Srrm4, Slit1, Sema3c, Sarm1, Olig2, Neurog2, Nes*
muscle organ development	*Adgrb1, Twist1, Myf5, Megf10, Hist1h1b*
spinal cord development	*Uncx, Gdnf, Hoxc10, Isl2, Olig2, Gdf7*
osteoblast differentiation	*Twist2, Twist1, Dlk1, Trp63, Tmem119, Sfrp2, Id4, Ddr2, Cebpa, Bmpr1b*

b

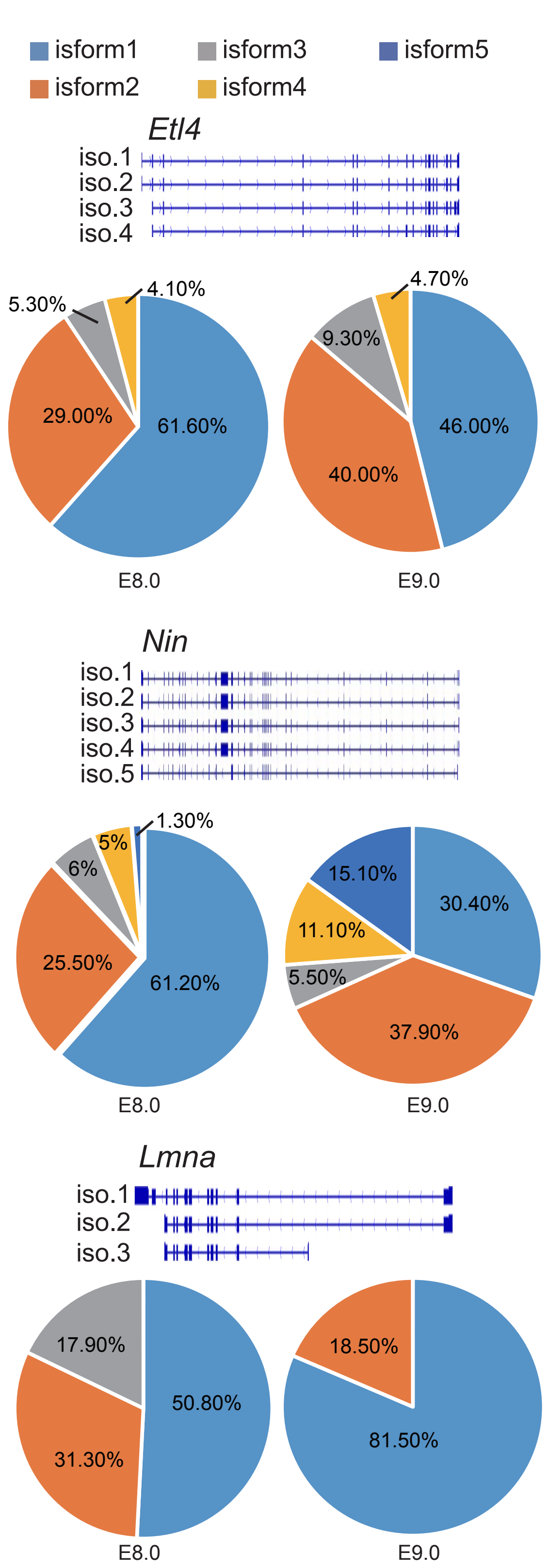

c

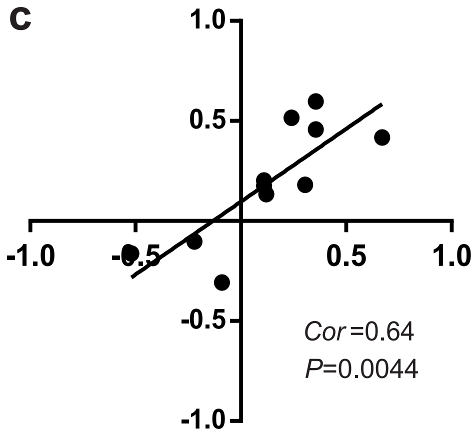

Transcriptional profiles of mouse early embryo organogenesis. a, Top, Heatmap visualization of differentially expressed genes (fold change >2, FDR <0.05; n=1185) between E9.0 (two embryos pooled per repeat) and E8.0 (five embryos pooled per repeat) mouse embryos. Z-score-normalized FPKM values were calculated based on independent biological samples with distinct RNA-seq libraries. Bottom, listed in the table are representative genes related to mouse embryonic development that are up-regulated in E8.0 and E9.0 mouse embryos. **b,** Top, Transcriptional structures of highly expressed gene isoforms detected by RNA-seq. Bottom, pie chart showing the proportion of each isoform of indicated genes in different stages. Isoform expression was quantified by RNA-seq (in FPKM). iso, isoform. **c,** Correlation for ΔPSI scores between RNA-seq data and RT-PCR results. Pearson's correlation coefficient was use to find statistical relations. Cor, correlation.

Supplementary Figure 2

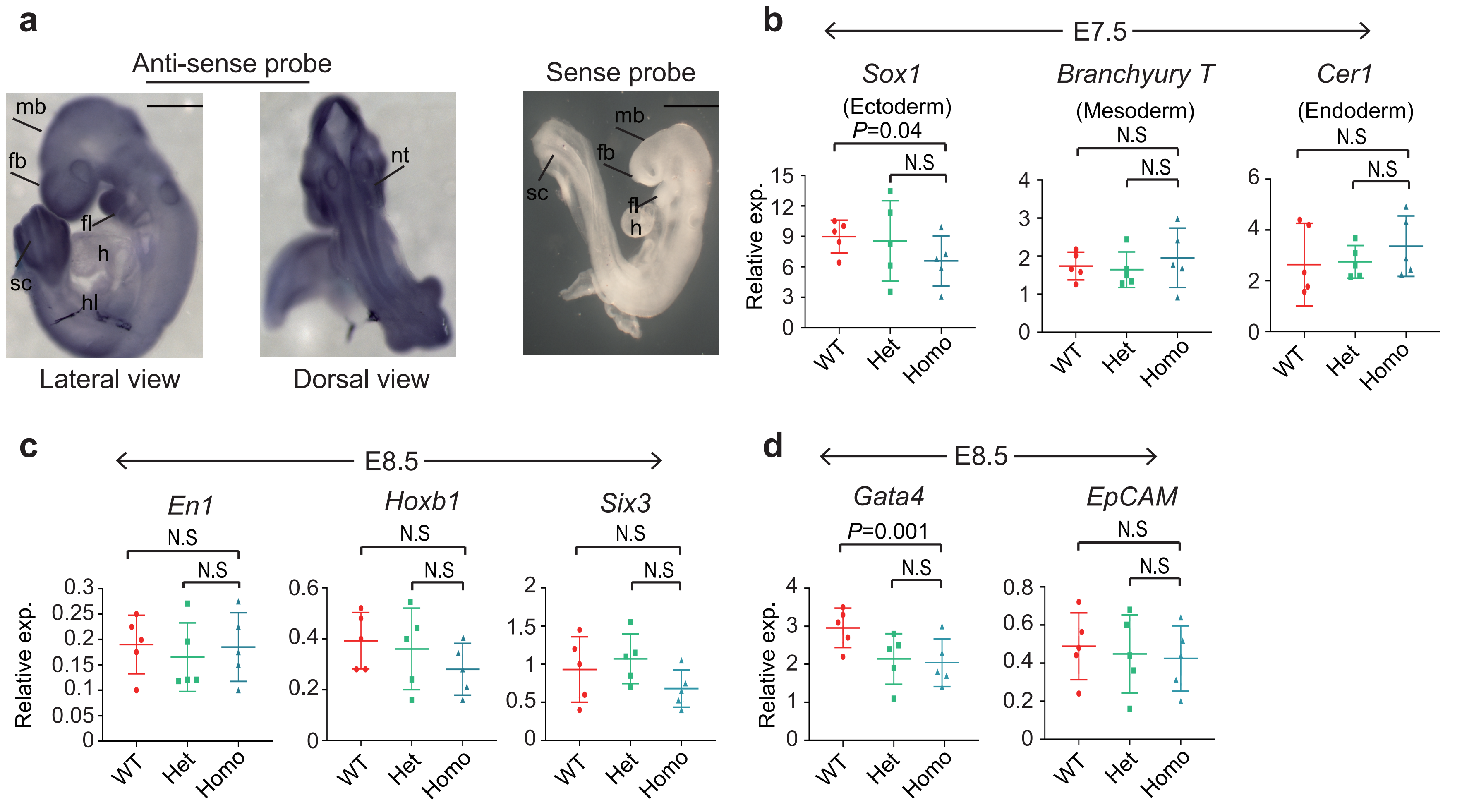

Strap deletion impairs the development of early mouse embryos. a, Whole mount in situ hybridization (WISH) analysis of B6 wild-type (WT) embryo for Strap mRNA. Left panel, lateral view of an embryo (E9.5) showing strong expression of Strap strongly in the fore brain (fb), mid brain (mb), spinal cord (sc), fore limb (fl), and hind limb (hl). Middle panel, dorsal view of the same embryo showing its expression in the region of the neural tube (nt). Right panel, a sense probe was used as a negative control. Scale bar, 300 um. **b-d,** qRT-PCR was used to quantify the relative mRNA levels in E7.5 (b) and E8.5 (c and d) embryos . P-values were based on unpaired two-tailed Student 's t- tests . Error bars indicate the mean ± SD from n = 5 biological replicates . Data represent one of three independent experiments . WT, wild type; Het, heterozygous ; Homo, homozygous ; N.S., not significant . Source data are provided as a Source Data file.

Supplementary Figure 3

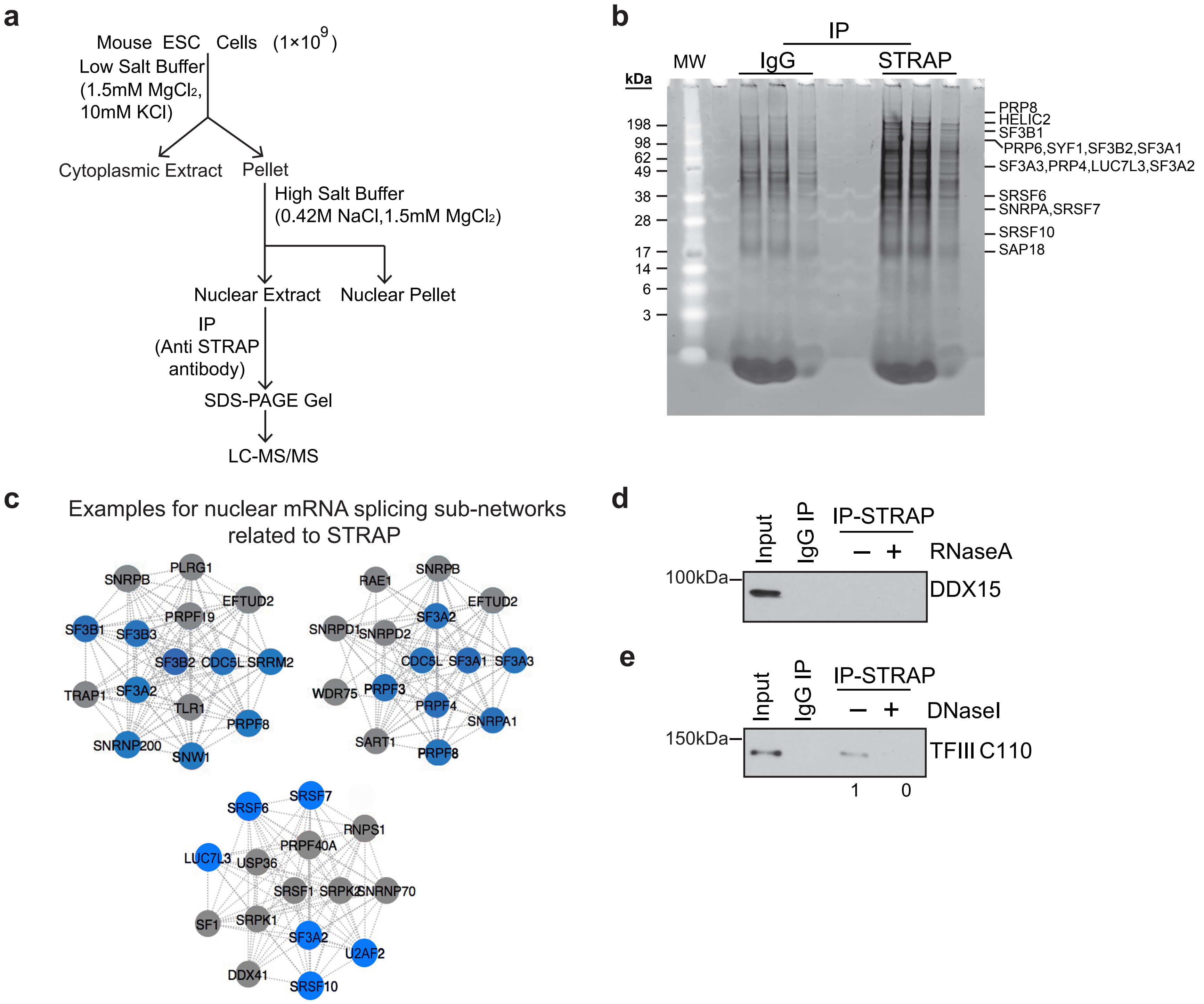

STRAP interacts with components of the spliceosome in ESCs. a, Schematic for the extraction of nuclear proteins in mouse ESCs for STRAP immunoprecipitation and LC-MS/MS. **b,** SyproRuby staining analysis of control IgG and anti-STRAP IPs (loaded in three lanes each) using NEs from mouse ESCs. Cellular nuclear partners of STRAP were determined by LC-MS/MS. Previously described, major spliceosome components also revealed in our experiment are designated on the right. **c,** Sub-networks for STRAP interaction with spliceosomal partners. All proteins indicated in blue were found to be bound with STRAP in our experiments. Proteins in grey are known members of spliceosome complexes. **d-e,** Immunoprecipitates were collected from NEs of ESCs after treatment either with RNase A or DNase I as indicated and then immunoblotted with antibodies against DDX-15 (d) or TFIIIC110 (e). For (d) and (e), 1% of lysates were loaded as input controls. The levels of each co-precipitated protein after treatment (relative to untreated control) are shown. The experiment was repeated twice, independently, with similar results. Source data are provided as a Source Data file.

Supplementary Figure 4

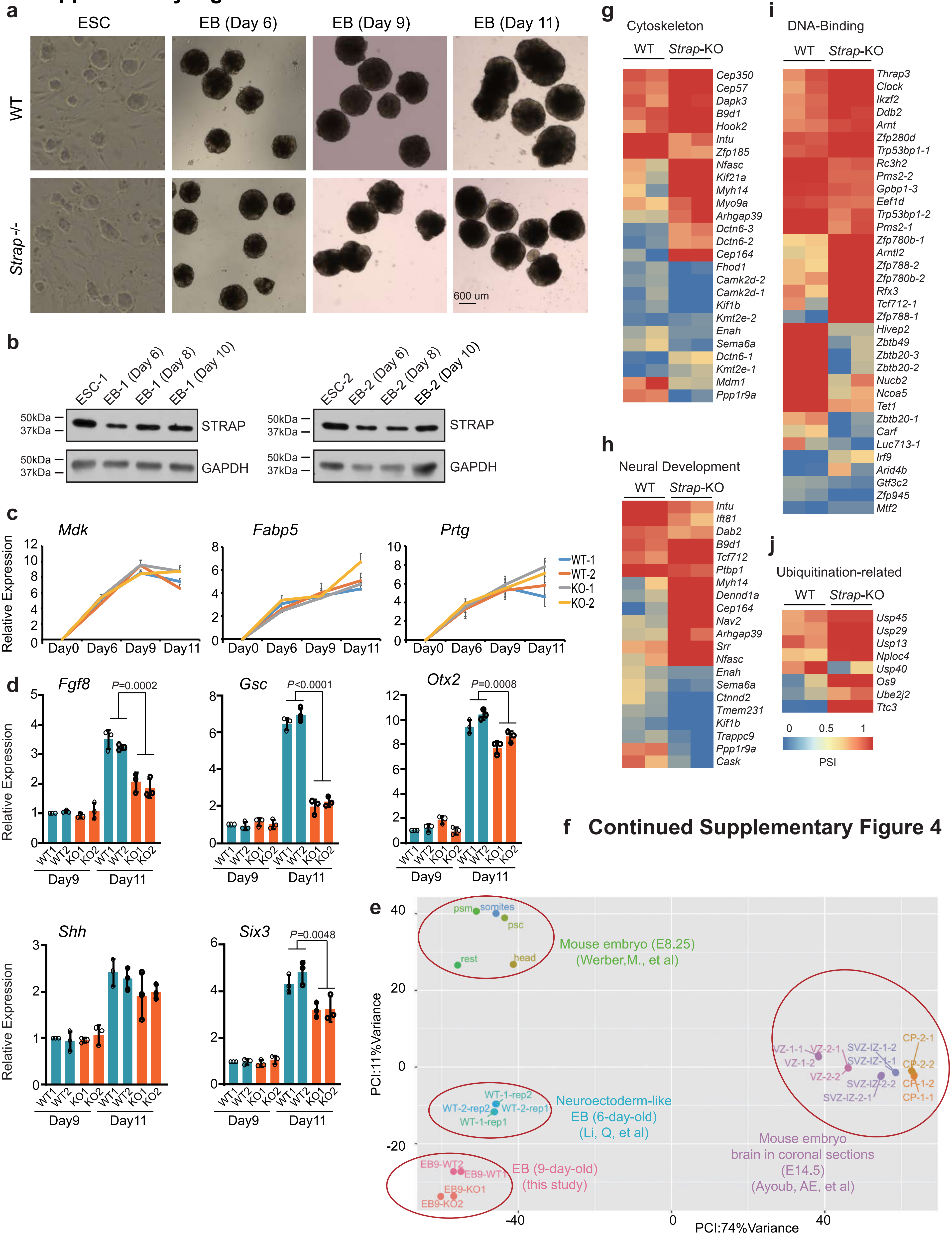

Continued Supplementary Figure 4

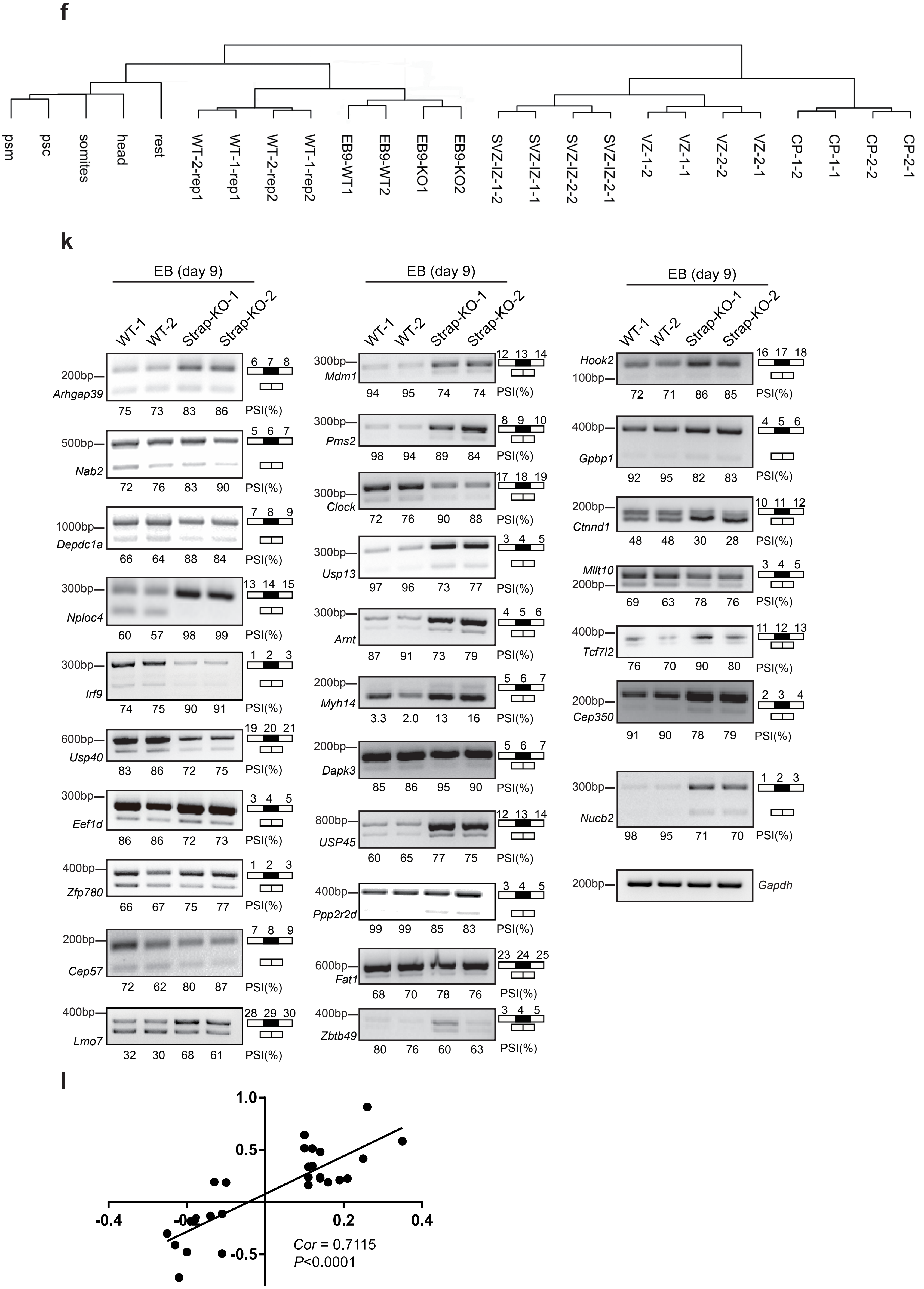

Numerous STRAP-mediated SE events are associated with EB neuroectoderm differentiation. a, Phase-contrast images of EB morphology during EB formation from Strap WT and KO ESCs at the indicated days. The experiment was repeated at least ten independent times with similar results. **b,** Western blots analyses of total cell lysates from indicated WT EBs were analyzed for STRAP expression. GAPDH was used as a loading control. **c,** qRT-PCR analysis of selected neuroectoderm markers in WT and Strap KO EBs at the indicated days. Values were normalized to Gapdh. **d,** Relative mRNA expression of early mouse brain development markers in WT and Strap KO EBs (9- and 11-day-old). Values were normalized to Gapdh. **e,** Principle-component analysis (PCA) for gene expression of neuroectoderm-like EBs33, mouse E14.5 brain34, mouse E8.25 tissue 35, and cultured EBs (this study) are shown. **f,** Hierarchy clustering of the four subgroups based on overall gene expression profiles (e). **g-j,** Splicing heatmaps showing the values of PSI for SE events between WT (n=2, biological replicates) and Strap-/- (n=2, biological replicates) EBs (9-day-old). Genes with significantly altered PSI values were selected. **k,** Validation by RT-PCR for indicated genes with SE events identified by rMATS. PSI values are shown below the gels. The information of target exons is shown in the right panel. Empty box: constitutive exon; black box: skipped exon. The experiment was repeated three independent times with similar results. **l,** Correlation for ∆PSI scores between RNA-seq data and RT-PCR results. Pearson's correlation coefficient was used to find statistical relationships. For (c) and (d), experiments were repeated two independent times with similar results; error bars indicate the mean ± SD from n=3 technical replicates. One-way ANOVA tests were used in (d). Source data are provided as a Source Data file.

Supplementary Figure 5

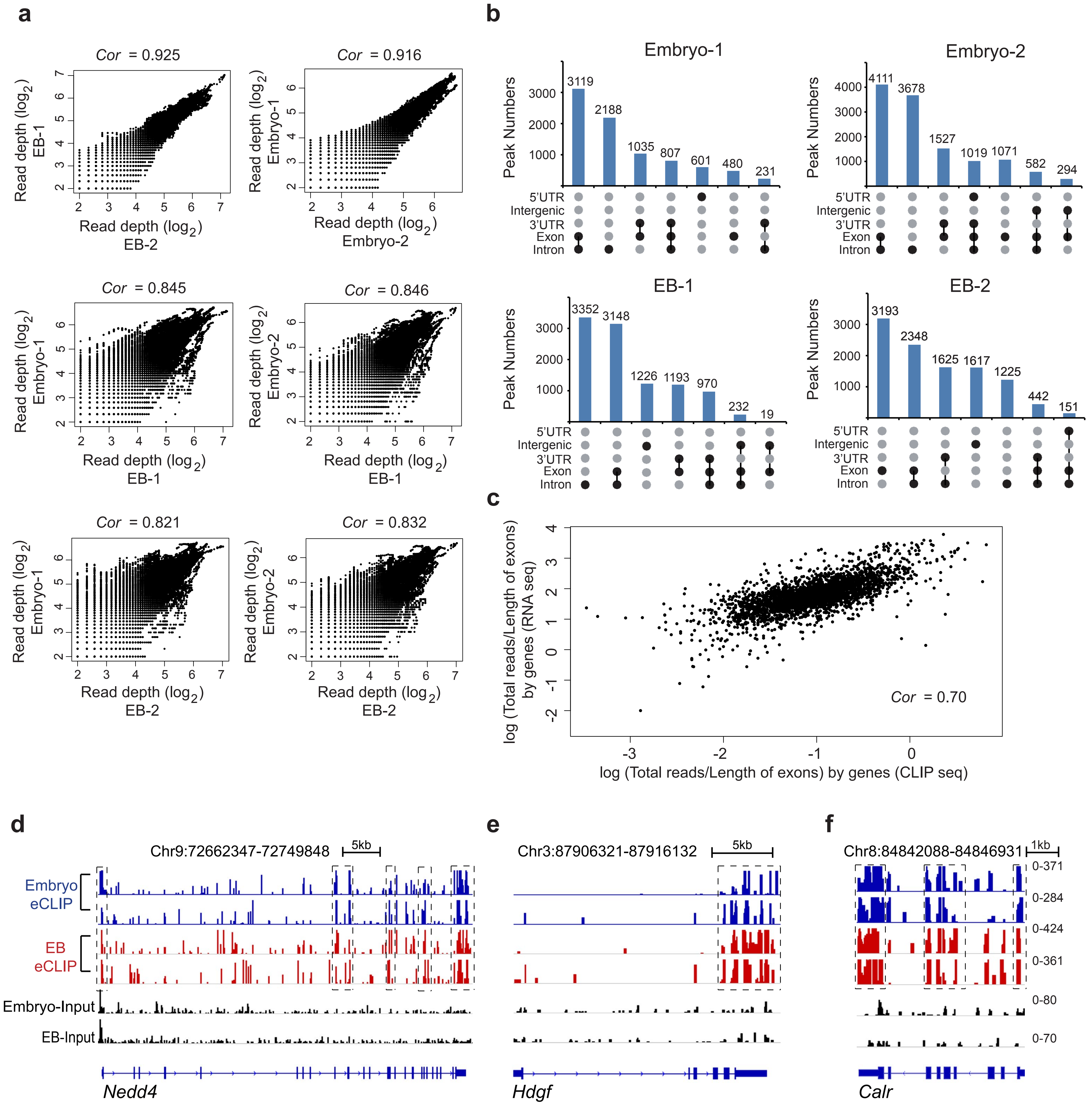

Characterization of STRAP eCLIP-seq data. a, Pairwise correlation of raw eCLIP read depth of coverage (the values of log2) between individual experiments . **b,** Distribution of STRAP eCLIP peaks in each sample across the mouse genome . The peak numbers were computationally counted by ChIPseeker Bioconductor packages . **c,** Pairwise comparison of read counts in given genes (n=3128) between RNA-seq and eCLIP-seq datasets . A representative plot was obtained from a WT EB sample . Pearson 's correlation coefficient (Cor) is indicated per plot . **d-f,** IGV viewer genome browser image of eCLIP signals in embryos and EB samples . Representative RNA-binding profiles of endogenous STRAP disproportionally located at intronic and exonic regions of Nedd4 (d), Hdgf (e), and Calr (f). The grey dot lined boxes highlight binding regions.

Supplementary Figure 6

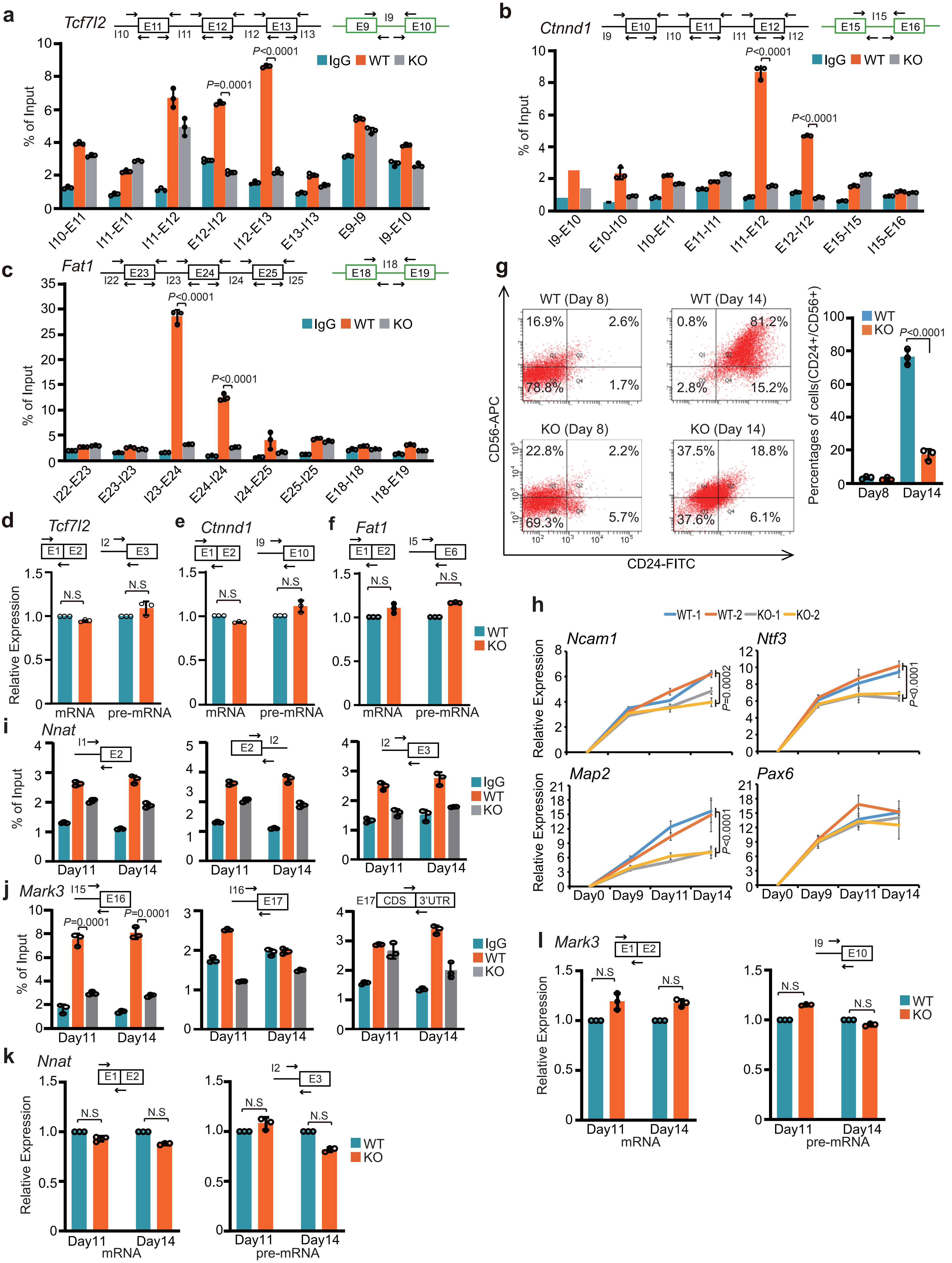

STRAP regulates skipped exons of Nnat and Mark 3 irrespective of their transcription during EB differentiation . a-c, RIP assay for STRAP binding to intron-exon or exon-intron junctions of Tcf7l2, Ctnnd1, and Fat1 in WT or Strap KO EBs (9-day -old). Schematic diagrams of targeted regions (black outlined box) and non -bound regions (green outlined box) with respective paired primers are shown at the top. **d-f,** qRT-PCR analyses of total mRNA and pre-mRNA for Tcf7l2 (d), Ctnnd1 (e), and Fat1 (f) in WT and Strap KO EBs (9-day-old). Values were normalized to Gapdh . Schematic diagrams of targeted regions with respective paired primers are shown at the top . **g,** Flow cytometry analysis of the percentages of combined neuronal markers (CD24+/CD 56+) in WT and Strap KO EBs are shown at the indicated days . Left, representative density plots show the data between groups at the indicated days. Right, bar plots show the percentages of CD24 +/CD+56 cells . Data were pooled from three independent experiments (n=3 per group) and error bars represent the mean ± SD **h,** qRT -PCR analysis of selected neural differentiation markers in WT or Strap KO EBs at the indicated days . Values were normalized to Gapdh . **i,j,** RIP assay for STRAP binding to intron -exon or exon -intron junctions of Nnat (i) and Mark3 (j) in WT or Strap-/- EBs (11- and 14-day-old). Schematic diagrams of targeted regions (black outlined box) with respective paired primers are shown at the top . **k,l,** qRT-PCR analysis of total mRNA and pre-mRNA for Nnat (k) and Mark3 (l) in WT and Strap KO EBs (11- and 14-day-old). Schematic diagrams of targeted regions (black outlined box) with respective paired primers are shown at the top . For all plots , except (g), error bars show the mean ± SD from n=3 technical replicates . All experiments , except (g), were repeated two independent times with similar results . P-values were determined by unpaired two- tailed t-test for (a-g) and (i-l). N.S, not significant. For (h), One-way ANOVA tests were used. Source data are provided as a Source Data file.

Supplementary Figure 7

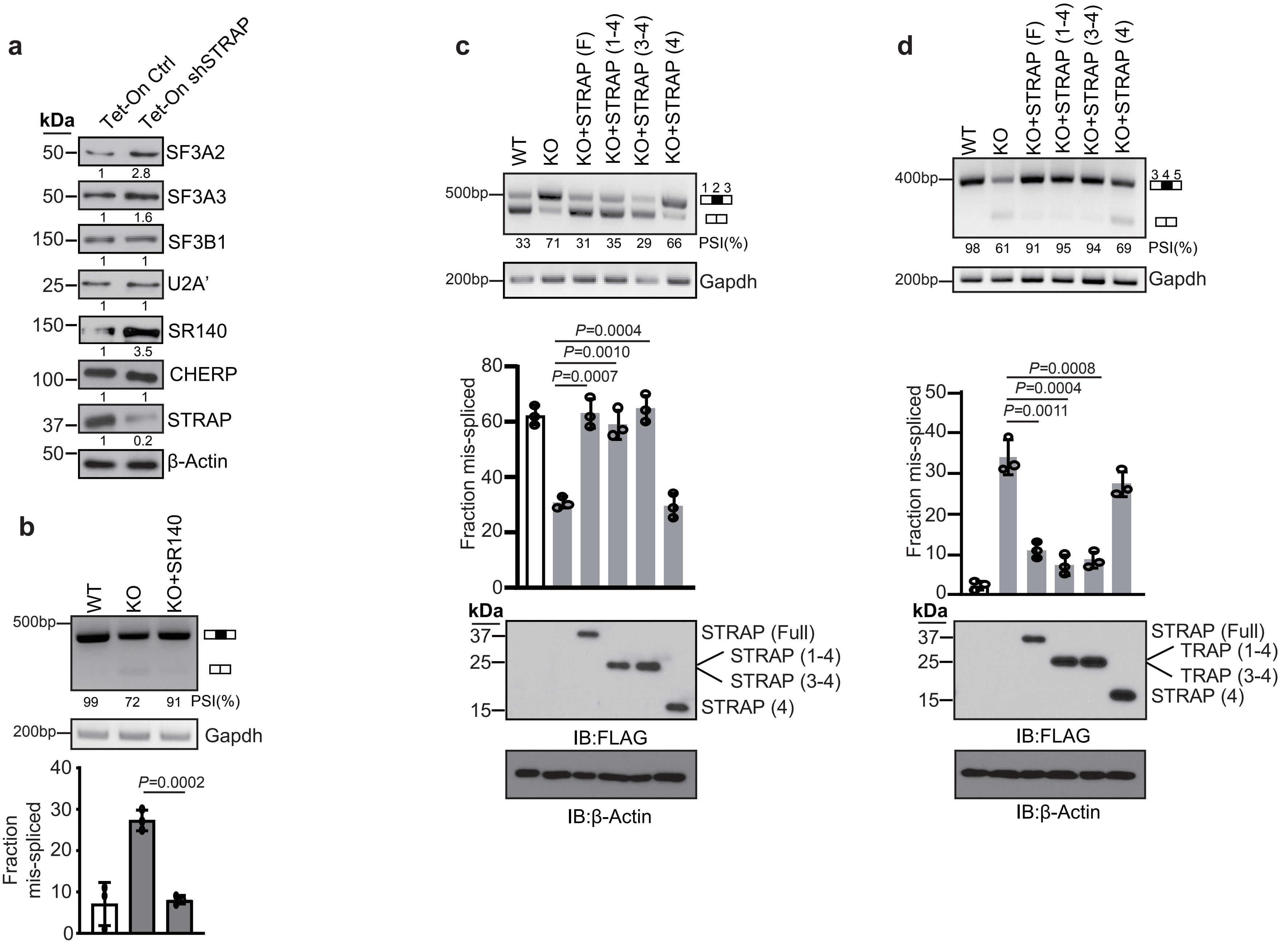

The WD-40 domain of STRAP is involved in splicing activity of certain alternative exons. a, Representative Western blots of various spliceosomal components in Tet-On E14 cell-derived EBs at day 14 are shown. EBs were derived from mouse E14 ESCs. Quantifications of protein levels are shown below the blots. ß-Actin was used as a loading control. The experiment was repeated three independent times with similar results. **b,** Rescue assay using the UPF3A mini-gene in WT and Strap KO MEFs. Cells were co-transfected with miniUPF3A and either an empty vector or a vector encoding SR 140. RT-PCR assays were performed to detect alternative exon inclusion in miniUSF3A. Quantifications of PSI values are shown below the gels. **c,d,** Gain-of-function assay using Nnat (c) and Ppp2r2d (d) mini-genes in WT and Strap KO MEFs. Indicated cells were co-transfected with mini-genes and either an empty vector or a vector encoding various Flag-tagged STRAP, as shown in Fig, 7(g). RT-PCR assays were performed to detect alternative exon inclusion in targeted exons. Quantifications of PSI values are shown below the gel. The Flag-tagged protein levels were assessed by Western blotting with ß-Actin as loading control. For (b), (c), and (d), the fractions of mis-splicing are pooled from three independent analyses (n=3). Error bars represent the mean ± SD. P-values were determined by unpaired two-tailed t-tests. Source data are provided as a Source Data file.

Supplementary Figure 8

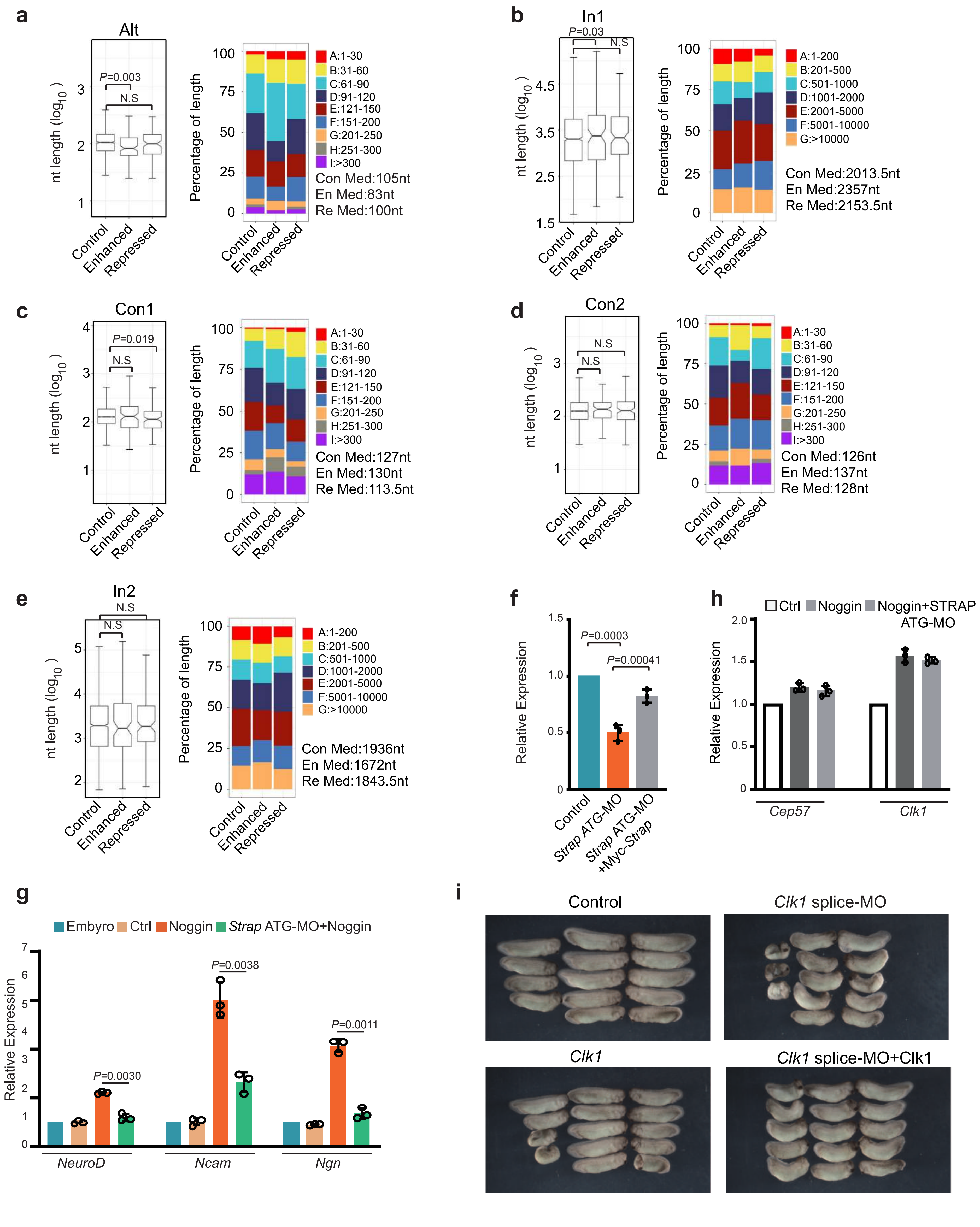

STRAP has a functional role in AS regulation during evolution. a-e, Alternative variants of different lengths affected by STRAP as revealed by rMATS analysis. Left, Box plot of cassette exon lengths and flanking intron lengths, comparing STRAP-dependent cases and exons without splicing changes. The lower and upper bounds of the box represent the 25th and 75th percentile of the distribution, respectively. The horizontal line in the box represents the median. The lower and upper whiskers show minima and maxima, respectively. The confidence interval around the median is shown as a notch. We defined 223 STRAP-dependent cassette exons (103 for enhanced and 120 for repressed). Alt, alternative exon; Con1, constitutive exon1; In1, intron 1; The P values were calculated with two-sided unpaired Wilcoxon tests. N.S: not significant. Right, bar height indicates percentage of the indicated exons or introns in each length category. Median values for each category are indicated at the side. Con: control; En: enhanced; Re: repressed; Med: median. **f,** Expression of mRNA for Strap analyzed by qRT-PCR in indicated Xenopus embryos at the neurula stage. **g,** qRT-PCR analyses of mRNA for neural differentiation markers in indicated Xenopus embryos at the neurula stage. **h,** qRT-PCR analyses of mRNA for Cep57 and Clk1 in indicated embryos at early tailbud stage. **i,** Clk1 splice-MO (50 ng) with or without Clk1 (0.5 ng) was injected into the dorsal animal regions of 4- to 8-cell stage embryos. The embryos were cultured until the tadpole stage and representative embryos are shown here. For (f) to (h), expression values were normalized to Histone2B and P-values were determined by unpaired two-tailed t-tests. Error bars show the mean ± SD from n=3 technical replicates. Experiments were conducted in three independent times with similar results. Source data are provided as a Source Data file.
