# Supplementary material for: STRAP regulates alternative splicing fidelity during lineage commitment of mouse embryonic stem cells
Source: Nat Commun. 2020 Nov 23;11:5941. doi: 10.1038/s41467-020-19698-6 (PMC7684319; doi:10.1038/s41467-020-19698-6)
Supplement: Supplementary file 3 — Reporting Summary [file 41467_2020_19698_MOESM3_ESM.pdf]

nature research

Corresponding author(s): Pran K. Datta, PhD

Last updated by author(s): Sep 18, 2020

Reporting Summary

Nature Research wishes to improve the reproducibility of the work that we publish. This form provides structure for consistency and transparency in reporting. For further information on Nature Research policies, see our Editorial Policies and the Editorial Policy Checklist.

Statistics

For all statistical analyses, confirm that the following items are present in the figure legend, table legend, main text, or Methods section.

n/a	Confirmed	
☐	☒	The exact sample size (*n*) for each experimental group/condition, given as a discrete number and unit of measurement
☐	☒	A statement on whether measurements were taken from distinct samples or whether the same sample was measured repeatedly
☐	☒	The statistical test(s) used AND whether they are one- or two-sided<br>*Only common tests should be described solely by name; describe more complex techniques in the Methods section.*
☒	☐	A description of all covariates tested
☐	☒	A description of any assumptions or corrections, such as tests of normality and adjustment for multiple comparisons
☐	☒	A full description of the statistical parameters including central tendency (e.g. means) or other basic estimates (e.g. regression coefficient) AND variation (e.g. standard deviation) or associated estimates of uncertainty (e.g. confidence intervals)
☐	☒	For null hypothesis testing, the test statistic (e.g. *F*, *t*, *r*) with confidence intervals, effect sizes, degrees of freedom and *P* value noted<br>*Give P values as exact values whenever suitable.*
☒	☐	For Bayesian analysis, information on the choice of priors and Markov chain Monte Carlo settings
☐	☒	For hierarchical and complex designs, identification of the appropriate level for tests and full reporting of outcomes
☐	☒	Estimates of effect sizes (e.g. Cohen's *d*, Pearson's *r*), indicating how they were calculated

Our web collection on statistics for biologists contains articles on many of the points above.

Software and code

Policy information about availability of computer code

Data collection

All software used in this study has been described in published literature.
For proteomics:
ReAdW (version 3.5.1) converted Xcalibur RAW files to MzXML. MzXML2Search (included in TPP v. 3.5) created mgf files.
For RNA-seq:
Cutadapt (version 2.2) was used for trimming primer adapters from raw FASTQ files. Sequencing reads were mapped to Gencode GRCm38 p4 Release M11 using STAR (version 2.5.2b).
For eCLIP-seq:
Sequencing reads were first trimmed using Cutadapt (version 2.2). The trimmed sequences were then mapped to the mouse RepBase to remove repetitive elements using STAR (version 2.5.2b). The unmapped reads from the STAR alignment were mapped to Gencode GRCm38 p4 Release M11 using STAR. Peaks were called on the reads that mapped to the mouse genome using MACS2 (version 1.4.2).

Data analysis

All parameters used to analyze data are described in methods section and custom scripts are available upon request.
For Prot eomics data:
The data was searched using SEQUEST(v.27 rec12. dta files), which was set for two maximum missed cleavages, a precursor mass window of 20ppm, trypsin digestion, variable modification C @ 57.0293, and M @ 15.9949. Searches were performed with a species specific subset of the UniRef100 database. The list of peptide IDs generated based on SEQUEST search results were filtered using Scaffold (version 3.0). STRAP binding-partners were ranked by COMPLEAT online tool (http://www.flyrnai.org>compleat).
For RNA sequencing data:
Transcript abundances were calculated using Cufflinks (version 2.2.1). Cuffmerge was then used to merge all of the transcript files from Cufflinks into one file. Following Cuffmerge, Cuffquant was used to quantify the transcript abundances, followed by differential gene expression using Cuffdiff. Differentially expressed genes with a P value < 0.01 as well as a log2 fold change > 1 were further analyzed. rMATS (version 3.2.5) created alternative splicing events files by using STAR alignments data. MAJIQ and Voila (https://biociphers.bitbucket.io/majiq/index.html) were used to detect, quantify and visualize local splicing variations (LSV) from the RNA-Seq data.
For eCLIP-seq analysis:

All reads with read depth greater than 3 were obtained using Samtools (version 1.3.1) and in-house Perl (version 5.26.3) scripts. The resulting log2 (Read depth) values were then plotted with the ggplot2 (version 3.2.1) package in R. eCLIP motif analysis was performed using HOMER software (http://homer.ucsd.edu/homer/ngs/peakMotifs.html). Based on genomic coordinates of each peak, the gene and transcript information was obtained through Mutalyzer (version 2.0.29 , https://mutalyzer.nl) and the corresponding read depth information was extracted from the BAM files using Samtools (version 1.3.1). eCLIP signals were visualized in track view using IGV (version 2.4.10).
For FACS analysis:
Becton Dickinson FACSDiva version 8.0 was used to analyze the positive cell population.
All of the statistical analysis were performed using R package (version3.3.3) or Prism version 7.0 (GraphPad).

For manuscripts utilizing custom algorithms or software that are central to the research but not yet described in published literature, software must be made available to editors and reviewers. We strongly encourage code deposition in a community repository (e.g. GitHub). See the Nature Research guidelines for submitting code & software for further information.

Data

Policy information about availability of data

All manuscripts must include a data availability statement. This statement should provide the following information, where applicable:

- Accession codes, unique identifiers, or web links for publicly available datasets
- A list of figures that have associated raw data
- A description of any restrictions on data availability

The mass spectrometry proteomics data have been deposited to the ProteomeXchange Consortium via PRIDE partner repository with the dataset identifier PXD015371. All sequencing data have been deposited in the GEO database at accession code GSE131474. Raw data for Figs 1e, 2c-d, 3c, 4e-g, 5a,5f, 6h-j,6l-o, 7a-h and 8d-f and Supplementary Figs 2b-d, 3b,3d-e,4b-d,4k,6a-l,7a-d, and 8a-h have been provided as Source data file. All other data supporting the findings of this study are available from the corresponding author on reasonable request.

Field-specific reporting

Please select the one below that is the best fit for your research. If you are not sure, read the appropriate sections before making your selection.

☒ Life sciences ☐ Behavioural & social sciences ☐ Ecological, evolutionary & environmental sciences

For a reference copy of the document with all sections, see nature.com/documents/nr-reporting-summary-flat.pdf

Life sciences study design

All studies must disclose on these points even when the disclosure is negative.

Sample size	In vitro sample sizes were partly limited based on number of available primary cell lines, but biological duplicates were performed at least 3 times independently for each cell line. They are sufficient to reproducibly discern biologically meaningful difference based on our previous study (doi: 10.1002/stem.2854). In vivo mice sample sizes (3 per group) were determined based on the previous study (doi:10.1016/j.scr.2009.02.002). In vivo Xenopus sample sizes were chosen based on prior studies (doi:10.1242/dev.168922; doi:10.1073/pnas.1711158114; doi: 10.1242/dev.111997), which validated the minimum number of Xenopus to determine a significant difference. This was determined at a range of 12-16 Xenopus embryos per group.
Data exclusions	No data were excluded from the analysis.
Replication	Experimental findings were reliably reproduced and results reported for 2-3 biological or technical replicates unless specified in the text.
Randomization	For teratomas experiment in age-matched female NON-SCID mice, animal were allocated for experiments randomly and were injected subcutaneously into the flank of mice with the same number of WT or STRAP KO mESCs. For Xenopus experiments, age-matched embryos were randomized into groups (12-16/group) for Morpholino or RNA injection or control group.
Blinding	Formal blinding was not performed during data collection and analysis. This was technically difficult for us because data collection by the same investigator and data analyses were performed using quantifiable parameters. For example, fluorescent signal values were used to measure the amount of DNA in each sample; and the values of percentage of positive neuronal cells were derived from FACSDiva using the same gating strategy. All experiments were performed multiple times to ensure reproducibility.

Reporting for specific materials, systems and methods

We require information from authors about some types of materials, experimental systems and methods used in many studies. Here, indicate whether each material, system or method listed is relevant to your study. If you are not sure if a list item applies to your research, read the appropriate section before selecting a response.

Materials & experimental systems

n/a	Involved in the study
☐	☒ Antibodies
☐	☒ Eukaryotic cell lines
☒	☐ Palaeontology and archaeology
☐	☒ Animals and other organisms
☒	☐ Human research participants
☒	☐ Clinical data
☒	☐ Dual use research of concern

Methods

n/a	Involved in the study
☒	☐ ChIP-seq
☐	☒ Flow cytometry
☒	☐ MRI-based neuroimaging

Antibodies

Antibodies used

STRAP antibody: IP,4 µg/mg lysate; eCLIP or RIP,5 µg/mg lysate; IF, 1:200, Bethyl, A304-735A.
STRAP antibody: WB, 1:1000, BD Transduction Laboratories, 611346, Lot#8128741.
SF3B1 antibody: IP, 4ug/mg lysate, Bethyl, A300-996A.
SNRPA antibody: WB, 1:1000, Santa Cruz, sc-376027, Clone B-12, Lot#E0316.
SR140 antibody: WB, 1:1000; IF,1:150, Santa Cruz, sc-398718, Clone E-3, Lot#E1816.
SF3A2 antibody: WB, 1:1000; IF,1:150, Santa Cruz, sc-390444, Clone A-3, Lot# E1116.
SF3B1 antibody: WB, 1:1000; IF,1:150, Santa Cruz, sc-514655, Clone B-3, Lot#E2471.
CHERP antibody: WB, 1:1000; IP, 1:500, Santa Cruz, sc-100650, Clone SS5, Lot#A1719.
SYF1 antibody: WB, 1:1000; IF,1:150, Santa Cruz, sc-271037, Clone C-9, Lot#E2571.
DDX15 antibody: WB, 1:1000; Santa Cruz, sc-271686, Clone E-6, Lot#D2816.
U2A' antibody: WB, 1:1000, Santa Cruz, sc-393804, Clone B-3, Lot#E0916.
HELIC2 antibody: WB, 1:1000, Santa Cruz, sc-393170, Clone G-9, Lot# E0416.
PRPF3 antibody: WB, 1:1000, Santa Cruz, sc-101130, Clone 42-N, Lot#F3017.
SF3A3 antibody: WB, 1:1000, Santa Cruz, sc-393673, Clone H-3, Lot#E1716.
TFIIIC110 antibody: WB, 1:500, Santa Cruz, sc-81406, Clone 2395C2a, Lot#G0817.
GAPDH antibody: WB, 1:1000, Cell Signaling, 2118S, Clone (14C10).
Beta-Actin antibody: WB, 1:10,000, Sigma, A5316.
HA antibody: WB, 1:1000; IP, 3ug /mg lysate, Bethyl, A190-208A.
Flag antibody: WB, 1:15,000, Sigma, F3165.
Alexa Fluor 488 goat anti-rabbit antibody: IF,1:200, Life Technologies, A-11008, Lot#1622775.
Alexa Fluor 555 goat anti-mouse antibody: IF,1:200, Life Technologies, A-21422, Lot#1837985.
CD24-FITC antibody: Flow, 2ul/test, MACS,#130-11-825, Clone REA743, Lot#1320010461.
CD56-APC antibody: Flow, 2ul/test, R&D systems, FAB7820A, Clone, 809220, Lot# ADIK0419101.
NESTIN antibody: IHC, 1:150, Cell signaling, #4760.
SMA antibody: IHC, 1:70, R&D systems, MAB 1420-SP.
AFP3 antibody: IHC, 1:200, R&D systems, MAB1368-SP.

Validation

Validation of antibodies in specific species and applications can be found with following links.
STRAP antibody: https://www.bethyl.com/product/A304-735A/STRAP+Antibody
STRAP antibody: https://www.bdbiosciences.com/eu/reagents/research/antibodies-buffers/cell-biology-reagents/cell-biology-antibodies/purified-mouse-anti-strap-22strap/p/611346
SF3B1 antibody: https://www.bethyl.com/product/A300-996A/SF3b155+SAP155+Antibody
SNRPA antibody: https://www.scbt.com/scbt/product/snrpa-antibody-b-12
SR140 antibody:https://www.scbt.com/scbt/product/sr140-antibody-e-3
SF3A2 antibody: https://www.scbt.com/scbt/product/sap-62-antibody-a-3
SF3B1 antibody: https://www.scbt.com/scbt/product/sap-155-antibody-b-3
CHERP antiboy: https://www.scbt.com/p/cherp-antibody-ss5
SYF1 antibody: https://www.scbt.com/scbt/product/hcnp-antibody-c-9
DDX15 antibody:https://www.scbt.com/scbt/product/ddx15-antibody-e-6
U2A' antibody: https://www.scbt.com/scbt/product/u2-snrnp-a-antibody-b-3
HELIC2 antibody: https://www.scbt.com/scbt/product/helic2-antibody-g-9
PRP3 antibody: https://www.scbt.com/scbt/product/prpf3-antibody-42-n
SF3A3 antibody: https://www.scbt.com/scbt/product/sap-61-antibody-h-3
TFIII3C antibody: https://www.scbt.com/p/tfiiic110-antibody-2395c2a
GAPDH antibody: https://www.cellsignal.com/products/primary-antibodies/gapdh-14c10-rabbit-mab/2118
Beta-Actin antibody: https://www.sigmaaldrich.com/catalog/product/sigma/a5316?lang=en&region=US
HA antibody: https://www.bethyl.com/product/A190-108A
FLAG antibody: https://www.sigmaaldrich.com/catalog/product/sigma/f3165?lang=en&region=US
Alexa Fluor 488 goat anti-rabbit antibody: https://www.thermofisher.com/antibody/product/Goat-anti-Rabbit-IgG-H-L-Cross-Adsorbed-Secondary-Antibody-Polyclonal/A-11008
Alexa Fluor 555 goat anti-mouse antibody: https://www.thermofisher.com/antibody/product/Goat-anti-Mouse-IgG-H-L-Cross-Adsorbed-Secondary-Antibody-Polyclonal/A-21422
CD24-FITC antibody: https://www.miltenyibiotec.com/CH-en/products/cd24-antibody-anti-mouse-reafinity-rea743.html#biotin:150-ug-in-1-ml
CD56-APC antibody: https://www.rndsystems.com/products/mouse-ncam-1-cd56-apc-conjugated-antibody-809220_fab7820a
NESTIN antibody: https://www.cellsignal.com/products/primary-antibodies/nestin-rat-401-mouse-mab/4760

SMA antibody: https://www.rndsystems.com/products/human-mouse-rat-alpha-smooth-muscle-actin-antibody-1a4_mab1420
APF3 antibody: https://www.rndsystems.com/products/human-mouse-alpha-fetoprotein-afp-antibody-189502_mab1368

Eukaryotic cell lines

Policy information about cell lines

Cell line source(s)	mouse ESC lines (WT and Strap-null) were derived from E3.5 blastocysts in our laboratory. MEF cell lines were derived from E10.5 WT and STRAP KO mouse embryos respectively. Mouse E14 cell line originated from ATCC.
Authentication	No cell line authentication was performed.
Mycoplasma contamination	All cell lines were tested for mycoplasma contamination and were confirmed negative.
Commonly misidentified lines (See ICLAC register)	No cell lines used in this study were found in the Register of Misidentified Cell Lines maintained by ICLAC (version 9).

Animals and other organisms

Policy information about studies involving animals; ARRIVE guidelines recommended for reporting animal research

Laboratory animals	For teratoma formation assay, 6-8 week-old female NON-SCID mice were purchased from Charles River Laboratories. To maintain Strap+/- strain, 10~12 week-old C57BL/6J mice (both male and female) were purchased from the Jackson Laboratory and bred in our facilities. 1-2 year-old Xenopus laevis frogs (both male and female) were purchased from Nasco.
Wild animals	No wild animals were used in this study.
Field-collected samples	This study did not involve field-collected samples.
Ethics oversight	All animal experiments were conducted in accordance with a protocol approved by Institutional Animal Care and Use Committee at University of Alabama at Birmingham.

Note that full information on the approval of the study protocol must also be provided in the manuscript.

Flow Cytometry

Plots

Confirm that:

☒ The axis labels state the marker and fluorochrome used (e.g. CD4-FITC).

☒ The axis scales are clearly visible. Include numbers along axes only for bottom left plot of group (a 'group' is an analysis of identical markers).

☐ All plots are contour plots with outliers or pseudocolor plots.

☒ A numerical value for number of cells or percentage (with statistics) is provided.

Methodology

Sample preparation	EB cells were digested and filtered to make a single cell suspension in staining buffer.
Instrument	Becton Dickinson LSRII
Software	Becton Dickinson FACSDiva version 8.0
Cell population abundance	No sorted cells were used in this study.
Gating strategy	Using a fluorescent antibody that is specific to the cells of interest, collect data on this fluorescent population. Gate on the fluorescent positive cells specific to the cell population of interest and then display the positive cells in the FSC vs SSC plot. Draw an FSC vs SSC gate around these fluorescent cells to outline the population of interest from the total cell population. This FSC vs SSC gate will then be used for acquisition and analysis of all samples.

☒ Tick this box to confirm that a figure exemplifying the gating strategy is provided in the Supplementary Information.
