# Supplementary material for: STRAP regulates alternative splicing fidelity during lineage commitment of mouse embryonic stem cells
Source: Nat Commun. 2020 Nov 23;11:5941. doi: 10.1038/s41467-020-19698-6 (PMC7684319; doi:10.1038/s41467-020-19698-6)
Supplement: Supplementary file 4 — Description of Additional Supplementary Files [file 41467_2020_19698_MOESM4_ESM.pdf]

`Description of Additional Supplementary Files

File Name: Supplementary Data 1
Description: Differentially expressed genes at the isoform level in mouse embryos at E8.0 and E9.0.

File Name: Supplementary Data 2
Description: AS profiles for mouse embryos in the transitional stage (from E8.0 to E9.0).

File Name: Supplementary Data 3
Description: The proteomic profile for STRAP-binding candidates.

File Name: Supplementary Data 4
Description: Transcriptome profiles for WT and Strap-KO ESCs and EBs (9-dayold).

File Name: Supplementary Data 5
Description: Comparison profiles of AS events between WT and Strap-KO EBs (9-day-old).

File Name: Supplementary Data 6
Description: STRAP-mediated AS profiles in the context of mouse ESCs or intestinal tissues.

File Name: Supplementary Data 7
Description: The LSVs profile upon deletion of STRAP in 9-day-old EBs.

File Name: Supplementary Data 8
Description: Profiles of STRAP high-confidence RNA-binding peaks.

File Name: Supplementary Data 9
Description: Profiles of STRAP-binding peaks at annotated alternative exons.

File Name: Supplementary Data 10
Description: Primers list for described experiments in this paper.
